# High Optical Response of Niobium-Doped WSe_2_-Layered Crystals

**DOI:** 10.3390/ma12071161

**Published:** 2019-04-10

**Authors:** Hung-Pin Hsu, Der-Yuh Lin, Jhin-Jhong Jheng, Pin-Cheng Lin, Tsung-Shine Ko

**Affiliations:** 1 Department of Electronic Engineering, Ming Chi University of Technology, New Taipei City 24301, Taiwan; hphsu@mail.mcut.edu.tw; 2 Department of Electronic Engineering, National Changhua University of Education, Changhua 50074, Taiwan; M0453002@mail.ncue.edu.tw (J.-J.J.); M0653013@mail.ncue.edu.tw (P.-C.L.); tsko@cc.ncue.edu.tw (T.-S.K.)

**Keywords:** 2D chalcogenides, photoconductivity, photoresponse

## Abstract

The optical properties of WSe_2_-layered crystals doped with 0.5% niobium (Nb) grown by the chemical vapor transport method were characterized by piezoreflectance (PzR), photoconductivity (PC) spectroscopy, frequency-dependent photocurrent, and time-resolved photoresponse. With the incorporation of 0.5% Nb, the WSe_2_ crystal showed slight blue shifts in the near band edge excitonic transitions and exhibited strongly enhanced photoresponsivity. Frequency-dependent photocurrent and time-resolved photoresponse were measured to explore the kinetic decay processes of carriers. Our results show the potential application of layered crystals for photodetection devices based on Nb-doped WSe_2_-layered crystals.

## 1. Introduction

Two-dimensional (2-D) semiconductors have attracted much attention since the discovery of graphene. Two-dimensional transition metal dichalcogenides (TMDCs) MX_2_ (M = transition metal; X = chalcogen) have shown their potential for optoelectronic and nanoelectronic applications [1,2,3,4,5]. Compared to graphene, layered TMDCs exhibit semiconductor, metallic and semi-metallic behaviors [6,7,8]. The bandgap properties can be changed from indirect to direct by varying the number of layers [9,10]. Other novel physical properties, such as spin-orbit band splitting and valleytronics [11,12,13,14], have been investigated as well. Top-gated transistors and high gain phototransistors based on the MoS_2_-layered semiconductor were also successfully developed [15,16]. MoS_2_ is well known to be a native n-type semiconductor, and p-doped MoS_2_ has also been achieved via intentional doping [17,18]. However, both n- and p-type semiconductors are needed for complementary digital logic electronic devices [19]. P-type behaviors have been observed in bulk and mechanically exfoliated monolayer and few-layer WSe_2_ [20]. Recently, theoretical and experimental studies reported modifications to optical [21,22], magnetic [23,24,25], and catalytic [26] properties induced by doping into TMDCs. The carrier type in layered TMDCs can also be modified by metal work-function engineering [27,28], electrostatic doping [29,30], surface functionalization [31,32], or charge transfer from physi-sorbed volatile molecules [33]. However, the replacement of the host atom with dopant is required because the covalent bonding inside the lattice is more stable in practical optoelectronics devices. Previously, it has been demonstrated that a certain amount of Nb (~0.5%) doping will generate acceptor states in MoS_2_, making the conduction type change from n-type to p-type [18]. However, few studies have been done on the use of Nb as a dopant in TMDCs such as WS_2_ (~0.55%) [34] or WSe_2_ (~0.17%) [35]. Impurity doping will create defect states that might change the carrier transport properties in 2-D materials. Hence, further exploration of the doping effects of 2-D TMDCs should be carried out.

A previous study revealed the enhanced photoresponsivity with Nb (~0.17%) doped into WSe_2_ [35]. It would be interesting to investigate the optical properties with the use of a higher Nb content in WSe_2_. Usually, high concentration doping is difficult to achieve in the initial stage. Hence, we chose a doping concentration of Nb of up to ~0.5%, which is similar to that used in other reported studies [18,34], and investigated the optical characteristics. In this work, we report the optical investigations of Nb-doped WSe_2_-layered crystals by piezoreflectance (PzR), photoconductivity (PC), spectroscopy, frequency-dependent photocurrent, and time-resolved photoresponse. The near band edge excitonic transitions were probed by PzR and PC spectra. The optical response was also carried out by frequency-dependent photocurrent and time-resolved photoresponse. We will report the optical measurements and discuss the possible mechanisms in this study.

## 2. Materials and Methods

The Nb-doped tungsten diselenide (WSe_2_) layered crystals were grown from 4N mixed elements (W: 99.99%; Se: 99.99%; and Nb: 99.99%) by the chemical-vapor transport (CVT) method. Chemical transport was achieved with ICl_3_ as the transport agent in an amount of about 0.3 g. The weight of the doping material was determined stoichiometrically to obtain a concentration of 0.5% Nb. The total charge used in the growth experiment was about 5 g. Prior to the crystal growth, the powdered compounds of the series were prepared from the elements through a reaction at 950 °C for 2 days in evacuated quartz ampoules. After cooling down to room temperature, the mixture was transferred into a three-zone furnace and slowly heated to 950 °C. In order to avoid any explosions due to strongly exothermic reactions between the mixed elements, slow heating was necessary. The growth temperature was about 850 °C with a temperature gradient of about 3 °C/cm and a growth time of ~15 days. Single crystals with layered structure were formed in silver-colored platelets with a thickness of ~20 um and lateral size of 2 mm × 3 mm.

For PzR measurements, the samples were glued on a 0.15 cm thick lead–zirconate–titanate (PZT) piezoelectric transducer driven by an 800 V_rms_ sinusoidal wave at ~200 Hz. The alternating expansion and contraction of the transducer subjected the sample to an alternating strain with a typical rms Δ*l*/*l* value of ~10^−5^. A 150 W tungsten-halogen lamp filtered by a PTI 0.25 m monochromator provided the incident monochromatic light. The reflected light was detected by a silicon photodetector (EG&G HUV-2000B, MD, USA). The dc output of the photodetector was maintained at a constant level throughout the experiment by an electronic servo mechanism with a variable neutral density filter. A dual-phase lock-in amplifier (Perkin-Elmer 7265, MA, USA) was used to measure the detected signals. For PC measurements, the spectra were measured as a function of the photon energy in the range from 1.0 to 2.3 eV by the probe beam with several µW, and the probe beam was chopped at ~10 Hz. For frequency-dependent photocurrent measurement, a voltage (5 V) was supplied by the source meter (Keithley 2400, OH, USA), and a 652 nm laser (~2 mW) was used as the excitation illumination. The dc photocurrent represented the steady state photocurrent at 0 Hz. Afterwards, an ac photocurrent was induced in the frequency range from 0.5 to 10 kHz. The time-resolved photoresponse signals were collected by a data acquisition (DAQ) device with time resolution of 1 μs.

## 3. Results and Discussion

**Figure 1:**
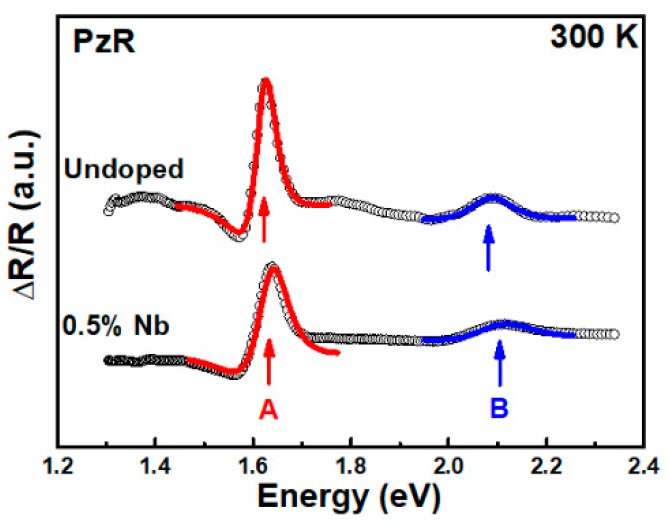
Experimental piezoreflectance (PzR) spectra of undoped and 0.5% Nb-doped WSe_2_-layered crystals at 300 K. The solid curves are the fits to the first derivative Lorentzian line shape. The obtained near band edge excitonic transitions A and B are indicated by arrows.

**Figure 2:**
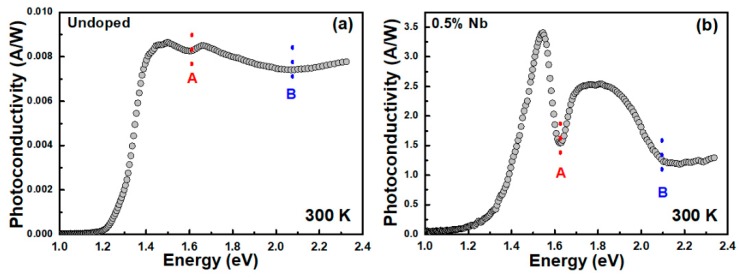
The photoconductivity (PC) spectra of (**a**) undoped and (**b**) 0.5% Nb-doped WSe_2_-layered crystals at 300 K. The excitonic transitions A and B are indicated by arrows.

Figure 1 shows the PzR spectra of undoped and 0.5% Nb-doped WSe_2_-layered crystals in the range from 1.3 to 2.35 eV at room temperature. As shown in the figure, two prominent near band edge excitonic transitions, A and B, of undoped and 0.5% Nb-doped WSe_2_ can be clearly observed. In order to determine the excitonic transitions from PzR spectra, we fit the experimental spectra with a theoretical line shape. The solid curves are the least-square fits to the first derivative Lorentzian line shape function [36,37]:

(1) ΔRR=Re∑j=1AjeiΦj(E−Ej+iΓj)−n

where *A_j_* and Φ*_j_* are the amplitude and phase of the line shape, *E_j_* and Γ*_j_* are the energy and broadening parameter of the excitonic transitions, and the value of *n* depends on the origin of the transitions. For the derivative functional form, *n* = 2 is appropriate for the bound states, such as excitons. The determined near band edge excitonic transitions of *E_j_* are denoted as A and B and are indicated by arrows in Figure 1. The obtained values of near band edge excitonic transitions A and B were 1.620 and 2.083 eV for undoped WSe_2_, whereas the excitonic transitions shifted to slightly higher energy levels with A and B showing values of 1.632 and 2.102 eV in Nb-doped WSe_2_ crystals. The PzR spectra revealed excitonic transitions with a slightly blue-shift following niobium incorporation. This blue-shift excitonic transition behavior was also observed in Nb-doped MoS_2_ by reflectance difference spectra [38]. The blue-shifted excitonic transitions in the present work might be due to the Burstein–Moss shift in the Nb-doped WSe_2_. Figure 2 shows the PC spectra for undoped and 0.5% Nb-doped WSe_2_-layered crystals. As shown in PC, we can observe that the WSe_2_ indirect band gap rose from ~1.2 eV. The other two features around 1.6 and 2.1 eV were attributed to the A and B excitonic transitions, respectively. It is shown that both the indirect band gap and direct excitonic transition can be measured by PC; however, the direct excitonic transition can be probed by PzR spectra with better accuracy for the determination of transition positions due to its derivative nature [39]. As compared with the undoped WSe_2_ sample, the photoresponsivity with Nb = 0.17% was enhanced by a factor of 10 [35]. However, the sample with Nb = 0.5% exhibited a much higher photoresponsivity intensity (~400 times) than the undoped one. In this study, the photoresponsivity can be increased significantly by incorporation of only a tiny amount of Nb (~0.5%) The value of undoped WSe_2_ (several mA/W) in this study is smaller than the value of few-layered WSe_2_ reported by Wang et al. (~600 mA/W) [40]. This result might be due to defects, which results in an inferior crystal quality and causes lower photoresponsivity in the PC measurement. However, the value of enhanced photoresponsivity in Nb =0.5 % WSe_2_ was ~3.5 A/W, which is comparable with previous studies based on multi-layered or mono-layer MoS_2_ photodetection devices [41,42]. The obtained photoresponsivity values for the Nb-doped WSe_2_ from this work and the MoS_2_ based photodetection devices from previous reports [40,41,42] are listed in Table 1.

**Figure 3:**
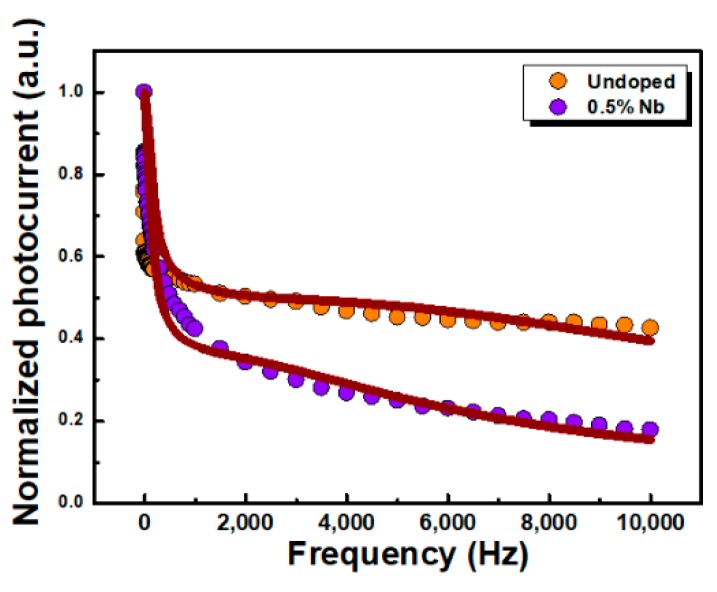
Normalized photoconductivity as a function of the frequency of undoped and 0.5% Nb-doped WSe_2_-layered crystals.

In order to understand the frequency response properties of undoped and 0.5% Nb-doped WSe_2_-layered crystals for the application in optoelectronic devices, the frequency-dependent photocurrent was also measured. To understand the carrier kinetics from the photoconductivity measurement, the frequency dependence of the photocurrent *I*_ac_/*I*_dc_ was measured, where *I*_ac_ is the ac component of the photocurrent and *I*_dc_ represents the steady state photocurrent. A light illumination source of 652 nm laser was used. Figure 3 illustrates the frequency-dependent photocurrent as a function of frequency for undoped and 0.5% Nb-doped layered crystals. It can be observed that the *I*_ac_/*I*_dc_ of Nb-doped crystal decreased faster than that of the undoped one as the frequency increased. The behavior of the frequency-dependent photocurrent can be described by the relation [43]

(2) $$I_{ac}/I_{dc}=k_{1}\times \tan h\left(\frac{1}{4{f\tau }_{1}}\right)+k_{2}\times \tan h\left(\frac{1}{4{f\tau }_{2}}\right)$$

where *K*_1_ and *K*_2_ are the amplitude coefficients. τ_1_ and τ_2_ are the carrier time constants of long and short time decay processes. The obtained values of the coefficients are listed in Table 2. In the frequency range from 0.5 to 10 kHz, the photocurrent decay in the Nb-doped crystal was composed of 67% long time and 33% short time processes. Compared to undoped WSe_2_ crystal, the proportion of long time constant decay process in Nb-doped crystal was larger than short time constant decay process. This might be attributed to additional trap states generated from the incorporation of niobium, which cause the longer decay process.

**Figure 4:**
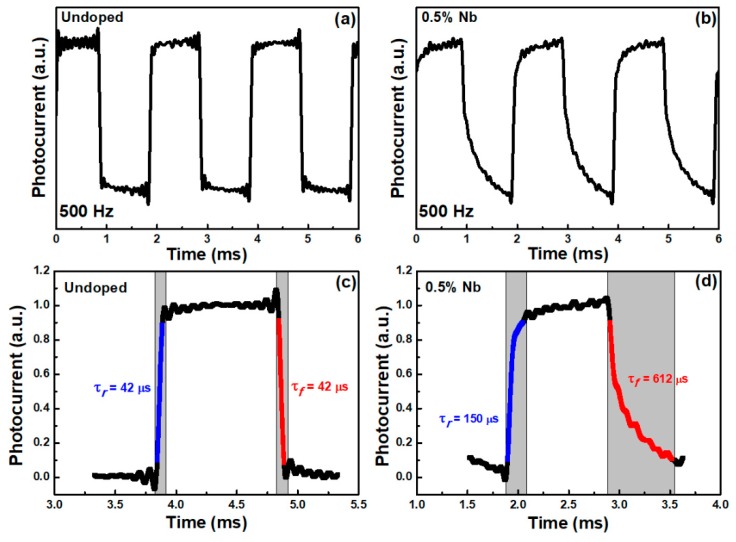
Time-resolved photoresponse of (**a**) undoped and (**b**) 0.5% Nb-doped WSe_2_-layered crystals at 500 Hz. The enlarged and normalized plots of (**c**) undoped and (**d**) 0.5% Nb-doped WSe_2_-layered crystals of one response cycle for calculating the rise and fall times.

To study the time-resolved photoresponse dynamics of undoped and Nb-doped WSe_2_-layered crystals, we applied ON/OFF light modulation at an incident light of 500 Hz and measured the rise and fall time constants. The time-resolved photoresponses of undoped and 0.5% Nb-doped layered crystals were further investigated and are shown in Figure 4a,b. The magnified and normalized plots of one response cycle are shown in Figure 4c,d. The speed response is characterized by the rise time (τ*_r_*) and the fall time (τ*_f_*). The rise time and fall time are defined as the time interval for the response rise from 10% to 90%, and the decay from 90% to 10% of the maximum photocurrent value, respectively [44]. The rise and fall time for undoped WSe_2_ was 42 μs. Further analysis of the 0.5% Nb-doped WSe_2_ revealed a larger rise time of 150 μs, as well as a larger fall time of 612 μs, which represents the slower photo response speed of Nb-doped crystal. It is noticed here that the timescales in the time-resolved photoresponse are different with the time constant determined by frequency-dependent photocurrent. This is because the rise and fall time originate from the combined contribution of both long and short time processes. This phenomenon is more pronounced with the incorporation of niobium which results in the rise and fall values being between long- and short-time constants. For the undoped WSe_2_, the response time is comparable with the previously reported TMDC values [45,46]. Nevertheless, the slower time response in Nb-doped crystal might be due to the influence of trap states induced by niobium impurity doping. The trap states could deteriorate the carriers’ transfer speed in the doped crystal [43]. The slower response speed that arises from trap states could also correlate with the enhanced photoresponsivity in PC measurement. The trap level produced by the doping atoms traps photoexcited carriers; then, the carriers could hop from trap level to energy band, continuously contributing to the photocurrent. For the sample with a Nb content of only 0.17%, the increased photoresponsivity was not as pronounced as in the sample with Nb = 0.5%. However, this mechanism makes the rise and fall response time longer in time-resolved measurement. The enhanced photoresponsivity accompany with slower time response in the Nb = 0.5%-doped sample provide a trade-off reference for optimizing the design of high performance photodetectors. The doping of niobium atoms creates trap states close to the energy band, which causes the enhancement of photoresponsivity as well as slowing down the response speed.

## 4. Conclusions

In conclusion, we studied optical properties, PzR, PC, frequency-dependent photocurrent, and time-resolved photoresponse, of 0.5% Nb-doped WSe_2_-layered crystals grown by the chemical vapor transport method. The near band edge excitonic transitions A and B were observed by PzR and PC spectra and showed a slight blue-shift of excitonic transitions following niobium incorporation. From the PC results, we found enhanced light illumination responsivity with the incorporation of Nb. The frequency dependence photocurrent results indicate that there is a larger portion of long decay processes in Nb-doped crystal. The time-resolved photoresponse analysis showed that the response time of Nb-doped crystal is larger than that of undoped crystal. Both the frequency dependence photocurrent and the time-resolved photoresponse investigation revealed lower response dynamics with the doping process, which was attributed to the additional trap states that result from the presence of niobium atoms. These results could be helpful for the optimization of photodetection applications based on TMDC-layered semiconductors.

## Figures and Tables

**Table 1:** Comparison of photoresponsivity for the WSe_2_ and MoS_2_ based photodetection devices.

Structure	Thickness	Photoresponsivity	Ref.
WSe_2_ (undoped)	bulk	8 mAW^−1^	this work
WSe_2_ (0.5% Nb)	bulk	3.5 AW^−1^	this work
WSe_2_ phototransistor	few-layers	600 mAW^−1^	[40]
MoS_2_/Si heterojunction	multi-layers	8.75 AW^−1^	[41]
MoS_2_ phototransistor	mono-layer	1 AW^−1^	[42]

**Table 2:** The obtained values of coefficients from the least-square fits to Equation (2) for undoped and 0.5% Nb-doped WSe_2_-layered crystals.

Specimen	*K*_1_	τ_1_ (ms)	*K*_2_	τ_2_ (μs)
WSe_2_ (undoped)	0.52	2.5	0.48	22
WSe_2_ (0.5% Nb)	0.67	3.1	0.33	51
